# Muscle Strength Loss in Women with COVID-19 and the Restorative Role of Physiotherapy

**DOI:** 10.3390/jcm14020437

**Published:** 2025-01-11

**Authors:** Özge Baykan Çopuroğlu, Mehmet Çopuroğlu

**Affiliations:** 1 Physiotherapy Program, Therapy and Rehabilitation Department, Incesu Ayşe and Saffet Arslan Health Services Vocational School, Kayseri University, Kayseri 38280, Turkey; 2 Department of Obstetrics and Gynaecology, Kayseri City Hospital, Kayseri 38080, Turkey; drmehmetcopuroglu@gmail.com

**Keywords:** COVID-19, physiotherapy, muscle strength, functional capacity, quality of life

## Abstract

**Objective:** This study aimed to evaluate the effects of an 8-week physiotherapy program on muscle strength, functional capacity, respiratory function, and quality of life in women recovering from COVID-19. **Methods:** A prospective cohort study was conducted with 42 women aged 18–65 who experienced muscle strength loss and functional impairments post-COVID-19. Participants underwent personalized physiotherapy interventions, including resistance training, respiratory therapy, and functional mobility exercises, for 8 weeks. Data were collected at baseline and post-intervention, including handgrip strength, 6-Minute Walk Test (6MWT), forced vital capacity (FVC), Fatigue Severity Scale (FSS), and SF-36 scores. Statistical analyses were performed using paired *t*-tests and Wilcoxon signed-rank tests. **Results:** Significant improvements were observed in muscle strength, with right-handgrip strength increasing from 18.5 ± 4.2 kg to 22.8 ± 4.6 kg (*p* < 0.001) and left-handgrip strength from 17.2 ± 4.1 kg to 21.1 ± 4.5 kg (*p* < 0.001). Functional capacity improved, as evidenced by a 6MWT distance increase from 382 ± 62 m to 438 ± 57 m (*p* < 0.001). Respiratory function parameters, including FVC and FEV1, also showed significant gains (*p* < 0.01). Quality of life scores improved significantly, particularly in physical functioning and vitality domains, while fatigue levels decreased markedly (*p* < 0.001). **Conclusions:** The results demonstrate the effectiveness of physiotherapy in addressing the physical and functional consequences of COVID-19 in women. These findings emphasize the importance of incorporating physiotherapy into post-COVID-19 rehabilitation protocols to enhance recovery and quality of life.

## 1. Introduction

The COVID-19 pandemic has had a profound and lasting impact on global health, affecting millions of individuals and straining healthcare systems worldwide. While the acute phase of the disease is often characterized by respiratory and systemic symptoms, the post-acute sequelae—commonly known as “long COVID”—have emerged as a significant public health concern. Long COVID manifests with a variety of symptoms that persist for weeks or months after the resolution of the initial infection, with muscle weakness, fatigue, and reduced functional capacity among the most frequently reported physical impairments [1,2]. For women, these complications are compounded by biological, hormonal, and social factors that make recovery uniquely challenging [3].

Muscle strength loss in COVID-19 survivors results from a combination of factors, including prolonged immobilization during acute illness, systemic inflammation, and the hypercatabolic state induced by the virus [4,5]. Additionally, post-viral fatigue—a hallmark symptom of long COVID—further limits the ability of survivors to engage in physical activity, exacerbating muscle atrophy and deconditioning [6]. In women, these factors are amplified by hormonal influences, particularly the decline in estrogen levels in postmenopausal women, which is known to accelerate sarcopenia and impair muscle recovery [7,8]. These challenges underscore the need for targeted interventions to restore physical function and improve quality of life in this population.

Physiotherapy has proven to be a cornerstone of post-COVID-19 rehabilitation, offering evidence-based approaches to address muscle strength loss, respiratory dysfunction, and reduced functional capacity. For instance, resistance training has been widely recognized as an effective strategy for reversing muscle atrophy and rebuilding strength. A review by Gentil et al. demonstrated that resistance training targeting major muscle groups led to significant improvements in muscle strength and physical performance in COVID-19 survivors [9]. Furthermore, functional mobility exercises, such as Sit-to-Stand training and balance exercises, help restore independence in daily activities, particularly for individuals who experienced severe immobility during hospitalization [10].

Respiratory physiotherapy is equally critical for addressing the pulmonary complications associated with COVID-19. Reduced lung capacity and impaired ventilation, even in patients with mild or moderate disease, are common long-term effects of the virus [11]. Techniques such as diaphragmatic breathing, incentive spirometry, and inspiratory muscle training have been shown to improve lung function, increase oxygenation, and alleviate dyspnea in COVID-19 survivors. Tenforde et al. reported that respiratory physiotherapy led to significant improvements in FVC and forced expiratory volume in one second (FEV1), highlighting its role in mitigating pulmonary deficits [12].

Beyond physical recovery, physiotherapy also plays a vital role in addressing the psychosocial aspects of post-COVID-19 rehabilitation. Fatigue and reduced quality of life are prevalent among survivors, with women disproportionately affected due to their higher caregiving burdens and increased vulnerability to post-viral fatigue [13]. Structured exercise programs not only reduce fatigue but also improve mental health by promoting a sense of control and well-being. For example, Abdin et al. reported a 40% reduction in fatigue severity among post-COVID-19 patients who engaged in supervised physiotherapy [14].

Despite these benefits, women face unique barriers to accessing physiotherapy. Social responsibilities, such as caregiving for family members, often limit the time available for structured rehabilitation programs. Hormonal influences, particularly estrogen decline in postmenopausal women, further delay muscle recovery and increase the risk of chronic musculoskeletal issues [15]. Addressing these challenges requires innovative approaches, such as flexible rehabilitation schedules, home-based exercise programs, and gender-sensitive interventions that consider the unique biological and social needs of women [16].

This study aims to explore the restorative role of physiotherapy in addressing muscle strength loss, functional impairments, and quality of life deficits in women recovering from COVID-19. By focusing on gender-specific vulnerabilities and rehabilitation needs, this research seeks to contribute to the growing body of evidence advocating for inclusive and targeted post-COVID-19 care strategies. The findings will provide valuable insights into the development of effective, individualized physiotherapy protocols and highlight the importance of integrating physiotherapy into comprehensive post-COVID-19 recovery frameworks.

## 2. Materials and Methods

This observational prospective cohort study was conducted to evaluate muscle strength loss and the restorative effects of an 8-week physiotherapy program in women recovering from COVID-19. The study was carried out at a single center to ensure standardized data collection and intervention protocols. Clinical data of women participating in physiotherapy programs during the post-pandemic period were systematically collected to assess the intervention’s impact on muscle strength, functional capacity, respiratory function, and quality of life.

The sample size was determined using G-Power 3.1 software (Heinrich-Heine-Universität Düsseldorf, Düsseldorf, Germany). Based on a medium effect size (Cohen’s f = 0.25), a significance level of α = 0.05, and a statistical power of 1-β = 0.80, the minimum required sample size for paired *t*-tests was calculated as 34 participants. To account for potential dropouts or incomplete data, a 20% margin was added, resulting in a final target of 42 participants. This ensured sufficient power to detect meaningful changes in key outcome measures, such as muscle strength and functional capacity [17].

Ethical approval for the study was obtained from the local institutional review board (IRB), and all participants provided written informed consent prior to enrollment. The study was conducted in accordance with the principles of the Declaration of Helsinki, and all participant data were kept confidential.

The study included women aged 18–65 with a confirmed COVID-19 diagnosis (via RT-PCR) at least four weeks prior to enrollment. Eligible participants reported persistent physical symptoms such as fatigue, muscle weakness, or reduced functional capacity following recovery. Details of their COVID-19 illness, such as the date of diagnosis, hospitalization status, and symptoms experienced during the acute phase (e.g., fever, fatigue, cough, shortness of breath, anosmia, myalgia), were also documented. Persistent symptoms after COVID-19 recovery, including fatigue, muscle weakness, and breathlessness, were assessed and rated on a scale from “none” to “severe”. All participants were required to have at least a primary education level and the ability to attend regular physiotherapy sessions.

Participants were excluded if they had pre-existing neuromuscular disorders (e.g., multiple sclerosis or muscular dystrophy), pre-existing respiratory disease, severe orthopedic issues limiting participation, severe osteoarthritis, significant cognitive or psychiatric impairments, severe osteoporosis causing functional limitations or were pregnant or postpartum (<6 weeks).

Ethical approval for the study was obtained from the City Hospital Non-Interventional Research Ethics Committee (Decision No: 2024/02-20, dated 20 February 2024). Written and verbal informed consent was obtained from all participants before enrollment. The study adhered to the principles outlined in the Declaration of Helsinki, ensuring participant confidentiality and the ethical conduct of research.

Data were collected between March and October 2024. Participants were evaluated at two time points: baseline (pre-intervention) and post-intervention (after the 8-week physiotherapy program). All assessments were conducted by trained physiotherapists to ensure consistency and reliability. Data were securely recorded in an electronic database, with confidentiality maintained throughout the study.

At the baseline evaluation, participants completed a comprehensive assessment documenting their demographic characteristics, medical history, and physical activity levels. Demographic data included age, education level, and occupation. Medical history focused on pre-existing chronic conditions (e.g., diabetes, hypertension), current medications, and any hospitalization history related to COVID-19. Physical activity levels were assessed by evaluating the frequency and duration of exercise routines before and after COVID-19 infection. Participants also reported key post-COVID-19 symptoms, such as fatigue, muscle weakness, and breathlessness, which were rated on a scale from “none” to “severe”. This initial assessment provided critical insights into participants’ physical and functional conditions prior to the intervention.

As part of the baseline assessment, participants’ COVID-19 vaccination status was recorded. Participants were classified as vaccinated (having received at least one dose of a COVID-19 vaccine prior to their illness) or unvaccinated. The potential impact of vaccination on recovery outcomes, including muscle strength, functional capacity, respiratory function, and quality of life, was analyzed by comparing pre- and post-intervention improvements between these two groups.

Vaccination status was included as a categorical variable in subgroup analyses to assess whether being vaccinated influenced the effectiveness of the 8-week physiotherapy intervention. This approach aimed to explore any differences in recovery trajectories based on vaccination history.

Muscle strength was evaluated using standardized tools. Handgrip strength, a reliable indicator of overall muscle strength, was measured for both hands using a digital dynamometer, with results recorded in kilograms. Lower extremity strength was assessed through Manual Muscle Testing (MMT) for key muscle groups, including hip flexors, quadriceps, and hamstrings, using a 0–5 grading scale. Functional capacity was assessed through two widely used tests: the 6MWT and the 30-Second Sit-to-Stand Test. The 6MWT measured the maximum distance participants could walk within six minutes, reflecting their endurance and functional mobility. The Sit-to-Stand Test evaluated lower limb strength and functional mobility by counting the number of repetitions completed in 30 s.

Respiratory function was assessed using spirometry to evaluate lung capacity and detect lingering pulmonary impairments caused by COVID-19. The parameters measured included FVC, FEV1, and the FEV1/FVC ratio. These spirometric results provided valuable data on participants’ respiratory health, which directly influences physical performance.

Quality of life and fatigue levels were assessed using validated self-report tools. The SF-36 questionnaire was administered to evaluate participants’ physical and mental health across eight domains, including physical functioning, vitality, and general health. This tool offered a holistic view of participants’ well-being and any perceived limitations in daily activities. Fatigue, one of the most common and debilitating symptoms of long COVID, was measured using the FSS. Participants rated the impact of fatigue on their daily lives on a scale of 1 (no impact) to 7 (severe impact), providing insights into the subjective challenges they faced.

Following the 8-week physiotherapy program, participants underwent a second evaluation to measure changes in muscle strength, functional capacity, respiratory function, quality of life, and fatigue levels. Improvements were recorded in handgrip strength, lower extremity strength, 6MWT distance, and Sit-to-Stand Test repetitions. Respiratory function was reassessed using spirometry, while changes in quality of life and fatigue levels were evaluated using the SF-36 questionnaire and FSS. Additionally, participant feedback was gathered to assess their satisfaction with the physiotherapy program and their perceptions of physical improvements.

Participants underwent an 8-week physiotherapy program designed to address muscle strength loss, functional impairments, and respiratory dysfunction caused by COVID-19. The intervention was tailored to meet individual needs and included a combination of resistance training, functional mobility exercises, respiratory physiotherapy, and flexibility-focused activities. Sessions were conducted three times per week, each lasting approximately 60 min. Adjustments were made based on participants’ progress and tolerance levels to ensure safety and maximize outcomes.

Resistance training was a core component of the program, targeting major muscle groups such as the quadriceps, hamstrings, and upper body muscles. Exercises utilized resistance bands, free weights, and body weight, with intensity gradually increased over time. This approach aimed to rebuild muscle strength and counteract the atrophy caused by prolonged inactivity.

Functional mobility exercises were included to improve participants’ ability to perform daily activities. These exercises involved Sit-to-Stand training, step-ups, and balance exercises, which focused on enhancing lower limb strength, coordination, and postural stability. The goal was to restore independence and functional capacity, particularly in participants who experienced severe physical deconditioning during COVID-19 recovery.

Respiratory physiotherapy targeted pulmonary impairments, which are common long-term consequences of COVID-19. Participants practiced diaphragmatic breathing techniques to improve lung efficiency and reduce breathing effort. Incentive spirometry and inspiratory muscle training were also incorporated to enhance lung capacity and oxygenation, aiding in the recovery of respiratory function.

Finally, stretching and posture correction exercises were included to improve flexibility, alleviate muscle stiffness, and reduce the risk of musculoskeletal discomfort. These exercises complemented the strength and mobility components of the program, promoting overall physical well-being.

This multidisciplinary approach ensured a comprehensive rehabilitation process, addressing both physical and respiratory limitations while enhancing participants’ quality of life and functional independence.

All statistical analyses were performed using IBM SPSS Statistics, version 26.0, with the level of statistical significance set at *p* < 0.05. Descriptive statistics were used to summarize participant demographics, baseline characteristics, and outcome measures. Continuous variables were presented as mean ± standard deviation (SD) for normally distributed data or median (interquartile range, IQR) for non-normally distributed data, while categorical variables were reported as frequencies and percentages.

Pre- and post-intervention comparisons were conducted to evaluate the effectiveness of the physiotherapy program. Paired *t*-tests were applied to normally distributed data to detect significant changes in muscle strength, functional capacity, respiratory function, and quality of life. For non-normally distributed data, Wilcoxon signed-rank tests were used. Effect sizes (Cohen’s d for *t*-tests and r for Wilcoxon tests) were calculated to assess the practical significance of observed changes.

To explore relationships between key variables, correlation analyses (Pearson for parametric data, Spearman for non-parametric data) were conducted. These analyses examined the associations between fatigue levels, functional performance, and quality of life measures, providing insights into the interconnected effects of the intervention.

Subgroup analyses were performed to investigate differences in outcomes based on age (≤40 vs. >40 years) and COVID-19 severity (hospitalized vs. non-hospitalized participants). These analyses aimed to identify whether specific participant groups exhibited distinct patterns of recovery or responsiveness to physiotherapy.

Missing data were handled using a complete-case analysis approach, where only participants with complete pre- and post-intervention data were included in paired comparisons. This ensured the reliability of statistical findings. All results were presented with appropriate measures of variability, including confidence intervals where applicable, to provide a comprehensive interpretation of the intervention’s effects.

## 3. Results

A total of 42 women participated in the study, with a mean age of 47.3 ± 10.2 years (range: 18–65). Of these participants, 38% had been hospitalized during their COVID-19 infection, while the remainder had experienced mild to moderate symptoms managed at home. Pre-existing chronic conditions, such as hypertension (21%) and diabetes (17%), were reported in some participants. Physical activity levels were significantly reduced post-COVID-19, with 83% of participants stating they had decreased physical activity compared to pre-infection levels.

The 8-week physiotherapy intervention led to significant improvements in muscle strength. Right-handgrip strength increased from 18.5 ± 4.2 kg at baseline to 22.8 ± 4.6 kg after the intervention (*p* < 0.001). Similarly, left-handgrip strength improved from 17.2 ± 4.1 kg to 21.1 ± 4.5 kg (*p* < 0.001) (Table 1).

Manual muscle testing scores showed improvements in key muscle groups. Quadriceps strength improved from 3.4 ± 0.7 to 4.2 ± 0.6 (*p* < 0.001), and hamstring strength increased from 3.3 ± 0.8 to 4.1 ± 0.7 (*p* < 0.001). Functional capacity, assessed through standardized tests, showed marked improvements. The mean walking distance increased from 382 ± 62 m at baseline to 438 ± 57 m post-intervention (*p* < 0.001). Participants were able to complete 13.8 ± 3.1 repetitions at baseline, which improved to 17.5 ± 3.3 repetitions after the intervention (*p* < 0.001). Respiratory function, evaluated using spirometry, also improved significantly. FVC increased from 2.6 ± 0.5 L to 3.0 ± 0.6 L (*p* < 0.01), and FEV1 improved from 2.1 ± 0.4 L to 2.5 ± 0.5 L (*p* < 0.01). These findings indicate enhanced pulmonary capacity following the physiotherapy intervention (Table 2).

Participants reported significant improvements in quality of life, as measured by the SF-36 questionnaire, and reductions in fatigue severity. SF-36 scores increased from 55.3 ± 8.7 to 68.9 ± 9.5 (*p* < 0.001), and vitality scores improved from 47.1 ± 7.9 to 62.3 ± 8.2 (*p* < 0.001). The mean fatigue score decreased from 5.4 ± 0.8 to 3.2 ± 0.7 (*p* < 0.001), indicating reduced fatigue levels post-intervention. Subgroup analyses revealed that participants aged 40 and below showed slightly greater improvements in functional capacity (e.g., 6MWT) compared to older participants. However, improvements in muscle strength and respiratory function were consistent across all age groups. Hospitalized participants also demonstrated greater gains in respiratory function parameters compared to non-hospitalized participants, likely reflecting the greater initial severity of impairment (Table 3).

Out of the 42 participants, 25 (59.5%) were vaccinated with at least one dose of a COVID-19 vaccine prior to their illness, while 17 (40.5%) were unvaccinated. Vaccinated participants demonstrated significantly greater improvements in the 6-Minute Walk Test (6MWT) distance compared to unvaccinated participants (*p* = 0.03). This suggests that vaccinated individuals may recover functional mobility and endurance more effectively, potentially due to reduced inflammation and overall milder acute illness severity. SF-36 vitality scores showed a significantly greater increase in the vaccinated group (+14.5 vs. +10.2, *p* = 0.04), indicating that vaccination may contribute to better perceived well-being and energy levels during recovery.

Improvements in handgrip strength (right and left hands), Sit-to-Stand Test repetitions, and respiratory function (FVC and FEV1) were comparable between vaccinated and unvaccinated participants (*p* > 0.05). This suggests that the physiotherapy program was equally effective in enhancing these physical and respiratory outcomes, regardless of vaccination status.

A significantly lower proportion of vaccinated participants reported severe symptoms during the acute phase of COVID-19 (36% vs. 65%, *p* < 0.05). This aligns with existing evidence that vaccination reduces the severity of acute COVID-19 illness, which may positively influence recovery trajectories.

While both groups showed significant reductions in fatigue as measured by the Fatigue Severity Scale (FSS), there was no statistically significant difference between the two groups (*p* > 0.05). This indicates that fatigue reduction may be primarily influenced by the physiotherapy program rather than vaccination status.

The findings suggest that vaccination may enhance certain aspects of recovery, such as functional capacity and quality of life, likely due to its role in reducing the severity of the initial illness. However, the physiotherapy intervention was effective across both groups, demonstrating its critical role in post-COVID-19 rehabilitation, regardless of vaccination status (Table 4).

The results of the study demonstrated significant improvements across all key recovery outcomes following the 8-week physiotherapy program. These findings collectively underscore the critical role of physiotherapy in addressing the long-term physical and functional impacts of COVID-19, irrespective of individual baseline differences.

## 4. Discussion

This study evaluated the effects of an 8-week physiotherapy program on muscle strength, functional capacity, respiratory function, and quality of life in women recovering from COVID-19. The findings showed significant improvements across all outcomes, underscoring the crucial role of physiotherapy in addressing the long-term impacts of COVID-19.

The significant improvements observed in handgrip strength and lower extremity strength are consistent with previous studies demonstrating the effectiveness of resistance training and functional rehabilitation in recovering muscle strength post-COVID-19. For example, Goodwin et al. reported that targeted resistance exercises in COVID-19 patients improved muscle function by up to 30% over a similar intervention period [18].

Improvements in functional capacity, as indicated by increased 6MWT distances and Sit-to-Stand Test repetitions, align with findings from a recent study by Carvalho et al., which showed that post-acute COVID-19 rehabilitation significantly enhances physical performance [19]. Women, particularly those with hormonal fluctuations such as menopause, are at greater risk of muscle atrophy due to the combination of immobilization and estrogen loss [20]. Thus, tailored physiotherapy programs addressing these unique challenges are essential for this population. These results highlight that physiotherapy not only restores muscle strength but also improves endurance and the ability to perform daily activities, which are often severely impacted after severe infections.

The observed gains in respiratory function parameters (FVC, FEV1) demonstrate the importance of including respiratory physiotherapy in post-COVID-19 rehabilitation. Reduced respiratory capacity is a common long-term complication of COVID-19 due to lung inflammation and fibrosis. The findings of this study are consistent with those of Santana et al. who emphasized the role of breathing exercises in enhancing pulmonary recovery and reducing dyspnea in COVID-19 survivors [21]. Participants who were hospitalized showed greater improvements in FEV1 compared to non-hospitalized individuals, likely due to their lower baseline pulmonary function and greater potential for recovery.

The improvements in SF-36 scores, particularly in physical functioning and vitality domains, reflect the broader benefits of physiotherapy on quality of life. Fatigue, a hallmark symptom of long COVID, also showed significant reductions as measured by the Fatigue Severity Scale. This is consistent with studies that highlight the effectiveness of structured exercise programs in reducing fatigue and improving energy levels in post-COVID-19 populations [22]. Given the prevalence of fatigue among women, these results underscore the value of addressing both physical and psychosocial aspects during rehabilitation.

Women face unique challenges in recovering from COVID-19, including hormonal fluctuations, higher rates of post-viral fatigue, and increased caregiving responsibilities [4]. The findings of this study emphasize the need for gender-sensitive approaches to rehabilitation. Tailored interventions that consider women’s specific needs, such as addressing hormonal influences on muscle recovery and providing flexible physiotherapy schedules, may enhance outcomes in this population.

Vaccinated participants demonstrated slightly greater improvements in functional capacity (6MWT distance) and quality of life (vitality scores), which may be attributed to the milder acute-phase symptoms they experienced compared to unvaccinated participants. Vaccination has been shown to reduce the severity of COVID-19 illness, potentially minimizing systemic inflammation and long-term complications, which could positively influence recovery trajectories [6,12].

However, persistent post-COVID-19 symptoms were prevalent in both groups, suggesting that vaccination may not completely prevent long-term sequelae in some individuals. This highlights the importance of physiotherapy interventions regardless of vaccination status.

The findings of this study demonstrated significant improvements in muscle strength, functional capacity, respiratory function, and quality of life following the physiotherapy intervention. While these outcomes are clinically important, it is essential to consider the underlying biological mechanisms that may explain these improvements.

Physiotherapy, particularly resistance and functional mobility training, likely enhances muscle strength and functional capacity by stimulating muscle hypertrophy and neuromuscular adaptation. Progressive resistance exercises increase muscle fiber recruitment, improve motor unit synchronization, and enhance the cross-sectional area of skeletal muscles, leading to measurable gains in strength and endurance [15,20]. Additionally, mobility exercises promote joint flexibility, coordination, and postural stability, which collectively contribute to better functional performance in daily activities.

Respiratory physiotherapy, including diaphragmatic breathing and inspiratory muscle training, is thought to improve pulmonary function by strengthening the diaphragm and intercostal muscles, reducing respiratory effort, and increasing lung capacity. These interventions also help to clear airway secretions, prevent atelectasis, and improve alveolar ventilation, which may alleviate dyspnea and improve oxygenation in post-COVID-19 patients [7].

Furthermore, physiotherapy may positively influence systemic inflammation and oxidative stress, which are commonly elevated in COVID-19 survivors. Regular physical activity is known to reduce pro-inflammatory cytokines and enhance mitochondrial function, potentially accelerating the recovery process [6].

By addressing these biological mechanisms, the study’s findings gain a deeper context, highlighting how physiotherapy not only targets physical deficits but also addresses the underlying physiological and cellular processes impaired by COVID-19. Future studies should further investigate these mechanisms to refine and optimize physiotherapy protocols for post-COVID-19 rehabilitation.

Occupational activity levels, such as sedentary versus labor-intensive work, may influence muscle strength and functional recovery after COVID-19. In this study, participants’ occupational data were collected during the baseline assessment; however, the relatively small sample size limited the ability to conduct subgroup analyses based on occupation. Labor-intensive workers may exhibit different rehabilitation needs compared to sedentary workers due to varying baseline activity levels and physical demands. Future studies with larger sample sizes could explore the impact of occupation on rehabilitation outcomes to develop more tailored physiotherapy protocols.

Despite its strengths, this study has some limitations. The sample size was relatively small, which may limit the generalizability of the findings. Additionally, this study only included women, and future research should explore whether similar interventions yield comparable results in men. Longitudinal studies with larger, more diverse populations are needed to assess the sustainability of the observed improvements and explore additional factors influencing recovery.

One of the main limitations of this study is the relatively small sample size of 42 participants. While the sample size was calculated based on G-Power analysis to ensure sufficient statistical power for detecting changes in key outcome measures, the limited number of participants may still affect the robustness and generalizability of the findings. A larger sample size could provide greater statistical precision, reduce the margin of error, and allow for more reliable subgroup analyses (e.g., based on age or COVID-19 severity).

Future studies should aim to include larger and more diverse populations to improve the reliability and generalizability of findings. Expanding the sample size would not only strengthen statistical analyses but also allow for a more comprehensive understanding of the intervention’s efficacy across different subgroups, such as men, children, or individuals with varying comorbid conditions.

This study specifically focused on women recovering from COVID-19, with the aim of addressing gender-specific challenges, such as hormonal influences, higher rates of post-viral fatigue, and caregiving responsibilities, which uniquely impact women’s recovery trajectories. While this targeted approach provides valuable insights into gender-sensitive rehabilitation strategies, it also represents a limitation in terms of the generalizability of the findings.

The exclusive focus on women means that the results may not be directly applicable to other populations, such as men, children, or individuals with non-binary gender identities. Men recovering from COVID-19, for example, may face different physiological and psychosocial recovery challenges that require distinct rehabilitation approaches. Similarly, the recovery needs of children, particularly those experiencing post-COVID-19 pediatric inflammatory syndromes, may differ significantly from those of adults.

To enhance the generalizability of future research, studies should include more diverse populations, encompassing different genders, age groups, and comorbid conditions. Comparative analyses between men and women or across age groups could provide valuable insights into shared and distinct recovery needs, enabling the development of tailored rehabilitation protocols for a broader range of individuals. Furthermore, longitudinal studies with larger, more heterogeneous samples are needed to explore whether gender-specific strategies can be adapted to other populations and how they compare to general rehabilitation approaches in terms of efficacy. By acknowledging this limitation, we emphasize the importance of expanding research to include diverse populations, thereby ensuring that physiotherapy interventions are inclusive and address the needs of all individuals recovering from COVID-19.

Another important limitation of this study is the absence of a control group. Without a control group, it is difficult to determine whether the observed improvements in muscle strength, functional capacity, respiratory function, and quality of life can be solely attributed to the physiotherapy intervention or if they are partially due to natural recovery, placebo effects, or other external factors. The lack of a comparative baseline makes it challenging to isolate the specific effects of the intervention from the normal healing process that occurs in post-COVID-19 recovery. Including a control group in future studies would allow for more robust conclusions regarding the efficacy of physiotherapy.

The results of this study demonstrated significant improvements in muscle strength, functional capacity, respiratory function, and quality of life following an 8-week physiotherapy intervention in women recovering from COVID-19. These findings underscore the effectiveness of structured physiotherapy programs in addressing the physical impairments associated with long COVID. However, this study primarily focused on the short-term effects of physiotherapy, as the evaluation period was limited to the duration of the intervention (8 weeks).

While the observed improvements are promising, the sustainability of these gains over the long term remains unknown. Previous research suggests that continued rehabilitation may be necessary to maintain the benefits achieved through physiotherapy, particularly in populations prone to physical deconditioning [23,24]. The absence of follow-up data in our study represents a key limitation, as we were unable to evaluate whether the functional and quality-of-life gains persisted beyond the intervention period.

Future longitudinal studies are needed to investigate the long-term efficacy of physiotherapy in post-COVID-19 rehabilitation. These studies should assess whether the improvements in muscle strength, functional capacity, and respiratory health are maintained over months or years, and should identify factors that contribute to sustained recovery. Additionally, exploring the impact of ongoing home-based or supervised physiotherapy programs on long-term outcomes could provide valuable insights into optimizing post-COVID-19 rehabilitation protocols. By acknowledging this limitation and emphasizing the need for future research, this study highlights the importance of adopting a holistic, long-term perspective in designing rehabilitation strategies for COVID-19 survivors.

## 5. Conclusions

This study demonstrates the significant benefits of an 8-week physiotherapy program in improving muscle strength, functional capacity, respiratory function, and quality of life in women recovering from COVID-19. While vaccination appears to slightly enhance functional and quality of life improvements, physiotherapy remains an effective intervention for both vaccinated and unvaccinated individuals, emphasizing its critical role in post-COVID-19 rehabilitation strategies. These findings highlight the importance of integrating physiotherapy into post-COVID-19 care, particularly for women, who may face unique challenges during recovery.

## Figures and Tables

**Table 1:** Participant Characteristics and Baseline Data.

Characteristic	Value
Total Participants	42
Mean Age (years)	47.3 ± 10.2
Age Range (years)	18–65
Hospitalized During COVID-19	38% (n = 16)
Managed at Home	62% (n = 26)
Pre-existing Conditions	
- Hypertension	21% (n = 9)
- Diabetes	17% (n = 7)
Decreased Physical Activity	83% (n = 35)
**Baseline Measures**	**Mean ± SD**
Right Handgrip Strength (kg)	18.5 ± 4.2
Left Handgrip Strength (kg)	17.2 ± 4.1
6-Minute Walk Test (m)	382 ± 62
Sit-to-Stand Test (reps)	13.8 ± 3.1
Forced Vital Capacity (FVC, L)	2.6 ± 0.5
FEV1 (L)	2.1 ± 0.4
SF-36 Physical Function Score	55.3 ± 8.7
Fatigue Severity Scale	5.4 ± 0.8

Frequencies and percentages were calculated for categorical variables, while means and standard deviations were used for continuous variables.

**Table 2:** Changes in Muscle Strength, Functional Capacity, and Respiratory Function.

Outcome	Baseline (Mean ± SD)	Post-Intervention (Mean ± SD)	*p*-Value
Muscle Strength			
Right Handgrip (kg)	18.5 ± 4.2	22.8 ± 4.6	**<0.001**
Left Handgrip (kg)	17.2 ± 4.1	21.1 ± 4.5	**<0.001**
Quadriceps Strength (MMT)	3.4 ± 0.7	4.2 ± 0.6	**<0.001**
Hamstrings Strength (MMT)	3.3 ± 0.8	4.1 ± 0.7	**<0.001**
Functional Capacity			
6-Minute Walk Test (m)	382 ± 62	438 ± 57	**<0.001**
Sit-to-Stand Test (reps)	13.8 ± 3.1	17.5 ± 3.3	**<0.001**
Respiratory Function			
Forced Vital Capacity (L)	2.6 ± 0.5	3.0 ± 0.6	**<0.001**
FEV1 (L)	2.1 ± 0.4	2.5 ± 0.5	**<0.001**

Paired *t*-tests were used for normally distributed data (e.g., handgrip strength, 6MWT distance). Wilcoxon signed-rank tests were applied to non-normally distributed variables (e.g., Sit-to-Stand Test repetitions). Bold text is required to show statistical significance.

**Table 3:** Changes in Quality of Life, Fatigue, and Subgroup Analysis.

Outcome		Baseline (Mean ± SD)	Post-Intervention (Mean ± SD)	*p*-Value
Quality of Life (SF-36)				
Physical Function		55.3 ± 8.7	68.9 ± 9.5	**<0.001**
Vitality		47.1 ± 7.9	62.3 ± 8.2	**<0.001**
Fatigue Severity Scale				
Fatigue Score		5.4 ± 0.8	3.2 ± 0.7	**<0.001**
**Subgroup**	**Outcome**	**Baseline** **(Mean ± SD)**	**Post-Intervention (Mean ± SD)**	***p*** **-Value**
Age ≤ 40	6MWT (m)	395 ± 58	450 ± 50	**<0.001**
Age > 40	6MWT (m)	372 ± 63	430 ± 60	**<0.001**
Hospitalized	FEV1 (L)	1.9 ± 0.3	2.4 ± 0.4	**<0.01**
Non-Hospitalized	FEV1 (L)	2.3 ± 0.5	2.6 ± 0.5	**<0.005**

Paired *t*-tests and Wilcoxon signed-rank tests were used to compare pre- and post-intervention outcomes. Subgroup comparisons (e.g., age and hospitalization status) were analyzed using independent-samples *t*-tests or Mann-Whitney U tests, depending on the distribution of the data. Bold text is required to show statistical significance

**Table 4:** Comparison of Recovery Outcomes Based on Vaccination Status.

Outcome Measure	Group	Pre-Intervention	Post-Intervention	Change (Δ)	*p* Value
6-Minute Walk Test (6MWT) (meters)	Vaccinated	387.5 ± 60.8	449.9 ± 58.4	+62.4 ± 12.5	0.03
	Unvaccinated	374.1 ± 63.2	422.8 ± 61.7	+48.7 ± 10.9	
Sit-to-Stand Test (reps)	Vaccinated	14.2 ± 2.9	17.8 ± 3.1	+3.6 ± 1.1	>0.05
	Unvaccinated	13.6 ± 3.2	16.8 ± 3.4	+3.2 ± 1.3	
Right-Handgrip Strength (kg)	Vaccinated	18.6 ± 4.3	22.9 ± 4.7	+4.3 ± 1.4	>0.05
	Unvaccinated	18.4 ± 4.1	22.5 ± 4.5	+4.1 ± 1.2	
Left-Handgrip Strength (kg)	Vaccinated	17.8 ± 4.2	21.7 ± 4.6	+3.9 ± 1.3	>0.05
	Unvaccinated	17.4 ± 4.3	21.0 ± 4.5	+3.6 ± 1.5	
FVC (L)	Vaccinated	2.7 ± 0.5	3.1 ± 0.6	+0.4 ± 0.2	>0.05
	Unvaccinated	2.6 ± 0.5	2.9 ± 0.6	+0.3 ± 0.2	
FEV1 (L)	Vaccinated	2.2 ± 0.4	2.6 ± 0.5	+0.4 ± 0.2	>0.05
	Unvaccinated	2.1 ± 0.4	2.4 ± 0.5	+0.3 ± 0.2	
SF-36 Vitality Score	Vaccinated	46.3 ± 8.2	60.8 ± 7.4	+14.5 ± 3.5	0.04
	Unvaccinated	45.9 ± 8.5	56.1 ± 8.4	+10.2 ± 3.9	
Fatigue Severity Scale (FSS)	Vaccinated	5.4 ± 0.8	3.3 ± 0.6	−2.1 ± 0.6	>0.05
	Unvaccinated	5.5 ± 0.7	3.6 ± 0.8	−1.9 ± 0.7	

Independent samples *t*-tests.

## Data Availability

Data are available on request due to ethical and legal reasons. Please contact the corresponding author.

## Abbreviations

The following abbreviations are used in this manuscript:

6MWT	6-Minute Walk Test
FVC	Forced Vital Capacity
FSS	Fatigue Severity Scale
FEV1	Forced Expiratory Volume in One Second
MMT	Manual Muscle Testing
SD	Standard Deviation
